# Wartime women giving birth: Narratives of pregnancy and childbirth, Britain c. 1939–1960

**DOI:** 10.1016/j.shpsc.2013.11.007

**Published:** 2014-09

**Authors:** Angela Davis

**Affiliations:** Centre for the History of Medicine, Department of History, University of Warwick, Coventry CV4 7AL, United Kingdom

**Keywords:** Second World War, Britain, Maternity, Narrative

## Abstract

Women in Second World War Britain benefitted from measures to improve maternal and child health. Infant and maternal mortality rates continued to fall, new drugs became available, and efforts were made to improve the health of mothers and babies through the provision of subsidised milk and other foodstuffs. However, in return, women were also expected to contribute to the war effort through motherhood, and this reflected wider cultural ideas in the North Atlantic world in the first half of the twentieth century which equated maternity with military service. The aim of this article is to examine the interplay between narratives of birth and narratives of war in the accounts of maternity from women of the wartime generation. It will explore how the military-maternity analogy sheds light on women’s experiences of pregnancy and childbirth in Britain during the Second World War, whilst also considering maternity within women’s wider role as ‘domestic soldiers’, contributing to the war effort through their traditional work as housewives and mothers. In doing so, the article reveals the complexity of women’s narratives. It demonstrates that they do not simply conform to the ‘medical vs. social’ binary, but reflect the wider cultural context in which women gave birth. Women incorporated the dominant discourses of the period, namely those around war, into their accounts.

## Highlights

• Women’s maternity narratives are complex and multilayered.
• Wartime tropes were central to their birth stories.
• Their accounts reflect the association between maternity and military service.

## 1 Introduction

During the Second World War efforts to increase Britain’s population resulted in renewed attention being paid to maternal health.^1^ It was not the first time that the experience of war had encouraged concern with maternal and infant welfare. Ann Oakley argues that the Boer War 1899–1902 was a critical moment in the history of antenatal care by revealing what appeared to be a shockingly low standard of health among the male population recruited to fight in that war. This revelation forced political attention on the actual condition of the Empire’s citizens.^2^ Infant welfare was included in the campaign to improve physical efficiency.^3^ Jane Lewis posits that the concern to stop the wastage of infant life ‘became even more explicit during World War I.’^4^ The loss of population during the war increased awareness of the importance of infant mortality, and child and maternal welfare work was extended to include the antenatal period. When the Ministry of Health was created in 1919, one of its six departments was devoted to maternal and child welfare. Such state intervention was justified in terms of the national good and rational improvement.^5^

The outbreak of the Second World War in 1939 again heightened the value of children for the future of the country. According to Lewis, ‘Fears about not only the welfare but also the numbers of people increased.’^6^ Irvine Loudon has shown that in consequence of this renewed focus on children’s wellbeing measures were brought in that were considered too expensive or politically unacceptable in peacetime.^7^ The National Milk Scheme introduced in June 1940 made subsidised or free milk available to all pregnant women or nursing mothers. In 1942 the Vitamin Welfare Scheme was extended to include expectant and nursing mothers (and children under five), allowing them free or inexpensive orange juice, cod liver oil or vitamin A and D tablets. By 1943, seventy percent of those eligible were participating in the Milk Scheme; forty-three percent of those eligible took up the orange juice; thirty-four percent the vitamins and twenty-one percent the cod liver oil.^8^ In addition, the number of hospital maternity beds rose by fifty percent during the war, thus ensuring that the pre-war trend towards the hospitalisation of childbirth continued. By the end of the war a majority of births took place in an institution for the first time.^9^ Within this overall picture of wartime development there were some retrenchments. Jose Harris points out that ‘the wartime growth of some social services has to be set against the wartime collapse of others.’^10^ Nonetheless maternal and infant health improved during the middle decades of the twentieth century.^11^

The aim of this article is to examine how these transformations in the maternity services in Britain were experienced by women giving birth during the war years. Given that developments in provision and practice were particularly wide-ranging during the war with the wartime experiments serving as a precedent for the National Health Service (NHS), studying the wartime generation is a useful way of assessing how women experienced and articulated change in maternity care. The article will investigate the importance women placed upon changes in the availability of healthcare services (both as a result of the war and the introduction of the NHS) in their narratives. In addition the essay will consider how wartime pronatalism portrayed women as contributing to the war effort through their traditional role as housewives and mothers. It will explore how these discourses were employed in women’s stories and the interplay between narratives of birth and narratives of war in their accounts. Finally it will ask whether the military-maternity analogy can shed light on women’s experiences of pregnancy and childbirth in Britain during the Second World War, whilst also considering how wartime rhetoric about women’s roles as ‘domestic soldiers’ shaped wider discourses about maternity and motherhood.

### 1.1 ‘Domestic soldiers’ and the military-maternity analogy

In an essay entitled ‘The Matrix of War: Mothers and Heroes’, the novelist Nancy Huston highlighted the ‘striking equivalence’ between maternal and military service.^12^ The anthropologist Omi Morgenstern–Leissner terms this the ‘military-maternity analogy’.^13^ Indeed anthropologists such as Morgenstern–Leissner and Robbie E. Davis-Floyd have explored how childbirth, and particularly hospitalised birth, can be seen as a rite of passage for women that has its parallel in military service for men.^14^ Historians have also demonstrated the strength of this military-maternity analogy in Western countries in the first half of the twentieth century. For example Susan Grayzel has shown that in First World War France proponents of pronatalist arguments suggested the equivalence of mothering and soldiering in order to demonstrate the need for the protection of maternity. She argues that the concept of ‘mobilization’ signified an association between society’s preparation for war and for childbirth.^15^ Cornelie Usborne has drawn attention to similar arguments in Germany at this time which equated women’s sacrifice to the fatherland in bearing and raising childbirth as equal to men’s military service.^16^ Likewise Patricia Stokes asserts that in Weimar Germany there was a ‘widespread cultural trope that equated women’s ‘service’ in childbirth with men’s risking their lives in battle’,^17^ and Nazi pronatalism has been discussed by Gisela Bock.^18^ Such ideas had also crossed the Atlantic. Elizabeth Temkin has noted that wartime pronatalism served as fertile ground for the growth of a national health programme for mothers and infants in the United States. ‘In the rhetoric of the day, the family took on political significance as an integral component of national security. Mothering, in particular, was portrayed as part of the war effort.’^19^

British women during the Second World War were also called upon to contribute to the war effort in their traditional roles as mothers. Although those women who stepped into male roles have been remembered most prominently in accounts of women’s wartime work,^20^ the majority of women were still doing ‘women’s jobs’, either at home taking care of their families or in forms of employment such as nursing, shop or factory work.^21^ Women’s labour was in demand during the war; in December 1941 the government passed the National Service Act (No 2), which made provision for the conscription of women. However traditional gender assumptions remained. Indeed women were extolled to use their skills in mothering to aid the war effort through acting as foster mothers to evacuees, childminders for mothers engaged in war work or staffing war nurseries. Women’s domestic role was championed. In a radio broadcast in 1940 Lord Woolton, Minister of Food, addressed women directly: ‘It is to you, the housewives of Britain that I want to talk tonight … We have a job to do, together you and I, an immensely important war job. No uniforms, no parades, no drills, but a job wanting a lot of thinking and a lot of knowledge, too. We are the army that guards the Kitchen Front in this war.’^22^ The war meant that women’s traditional work took place in an entirely new context, though.^23^ In her diary for Mass Observation Nella Last wrote of her wanting to serve her country through her voluntary work as her son was doing in the army.^24^ She ‘vowed to be a soldier too’.’^25^ Indeed describing them as ‘domestic soldiers’, Jennifer Purcell explains that ‘Women became the vanguard in the People’s War.’^26^

## 2 Sources and approach

### 2.1 The Oxfordshire interviewees

The principal source material for the article are fifty oral history interviews I conducted with Oxfordshire women, born between 1912 and 1937, who had experienced the Second World War as children or adults and had their children either during the war or in the years soon after.^27^ They were members of the wartime generation. While the Oxfordshire interviewees originated from around the country (and some were born abroad), they were all living in Oxfordshire when they raised their children. However the interviewees were chosen to represent different types of location—rural, urban and suburban—namely the villages of Benson and Ewelme in south Oxfordshire; the Wychwood villages in west Oxfordshire; the twenty-four square miles near Banbury in north Oxfordshire covered by the *Country Planning* (1944) survey^28^; Oxford city centre; and the contrasting suburbs of Cowley and Florence Park in east Oxford and North Oxford and Summertown in north Oxford. In both Oxfordshire and the city of Oxford maternal mortality, infant mortality and perinatal mortality rates were generally below the national average. The rates for Oxford also tended to be lower than those for Oxfordshire. Oxfordshire was often at the forefront of developments in maternal care during these years, encouraged by the presence of the university and a large teaching hospital—there was the early provision of a family planning clinic in 1935 and a move to attaching health visitors to doctors’ practices in 1955.

The interviewees were principally found through community groups and social clubs and by personal recommendations. Kate Field argues this ‘snowballing’, where each respondent gives the name of another person to participate, is a particularly appropriate method for finding elderly respondents to a local study because it helps secure the trust of interviewees through being ‘recommended’ to them by their friends.^29^ The sample was self-selecting in that all the women had volunteered to be interviewed. However the aim was to construct a sample that represented both middle and working classes^30^ and a variety of educational backgrounds (from minimum-age school leavers to graduates) to see how locality, education and class influenced women’s experiences. The interviews were semi-structured, following the model described by Penny Summerfield,^31^ and were typically between one and two hours long. To address some of the ethical issues surrounding oral history all the potential respondents were informed in advance of the interview about the aims of the research. They therefore had the opportunity to make decisions about what they would choose to divulge prior to the interview which placed them in a more powerful position. This advance notification also prevented any difference in expectation between interviewer and interviewee. All interviewees were asked to sign consent forms at the end of the interview whereby they had the chance to specify any restrictions they wished to make on their contributions. Pseudonyms have been used to protect the interviewees’ privacy.

The accounts of the Oxfordshire interviewees will be seen in relation to other wartime women’s stories including autobiographies, contemporary Mass Observation survey material and oral history interviews undertaken for other collections. While these sources differ in their form and purpose from the interviews I conducted, they are also narratives of birth and war. Analysing the Oxfordshire interviewees alongside these additional accounts helps to consider the sample’s representativeness in terms of the stories they told, and also whether their experiences were indicative of wider social and medical trends. Indeed both types of source material (the oral history interviews and the other accounts) will be ‘read’ in these two ways—firstly in order to examine how women engage with cultural discourses in their accounts, but also to ‘explore’ the medical and social context of the time.

### 2.2 Oral history narratives

Within the field of oral history there has been a growing recognition of the significance of the narrative itself, beyond the ‘facts’ it contains. Lynn Abrams has gone so far as to argue that the term ‘narrative’ has become ubiquitous in oral history in recent years.^32^ Historians have come to appreciate that narrative is fundamental to the ways in which people recall the experience of their lives.^33^ Oral history narratives are not ‘out there’ waiting to be discovered, but are products of the interview process.^34^ Consequently collecting narratives is not simply about gathering evidence, but also about striving to understand how people feel about, interpret, remember, and represent their experiences within the context of the interview.^35^ People use narrative genres to structure their accounts.^36^ Broader cultural and literary emblems, as well as presentations of life and self, are woven into, and help shape, the stories people tell.^37^ Their narratives are also gendered. Reflecting upon this point Joan Sangster posits that, ‘Asking why and how women explain, rationalise and make sense of their past offers insight into the social and cultural patterns they faced, and the complex relationship between individual consciousness and culture.’^38^ Many of these same considerations also need to be applied to other forms of self-narratives and life-writing.^39^ Writing the histories of women’s (and men’s) subjectivities requires historians to engage with the ways in which lived experience is transformed into a coherent life narrative.

### 2.3 Narratives of health and illness

The value of the medical narrative as social and cultural evidence of health and illness has long been noted by both anthropologists and historians, but in recent years there has been a remarkable growth of interest in ‘illness narratives’. The number of published studies of patients’ and others’ accounts of illness, disability and other bodily phenomena has grown rapidly. Path-breaking studies, such as those by Arthur Kleinman, Elliot George Mishler and Arthur Frank,^40^ have helped to establish the study of illness narratives, and to construct it as a specialist domain of inquiry in its own right. The work of Kleinman in particular has placed ‘the illness experience’ at the centre of understanding health and medicine through demonstrating how ‘illness narratives edify us about how life problems are created, controlled, made meaningful’.^41^ Discussing how theories of oral history and illness experience both emphasise the importance of the individual account in interacting with and illuminating broader socio-historical trends, Kerry Davies stresses that examining the ways in which people negotiate, frame, and tell their stories is crucial to understanding their experiences.^42^ Cheryl Mattingley goes further, arguing that narrative is inherent to the patient/practitioner interrelationship. Stories are not only ‘after-the-fact accounts of experience, or cultural scripts which provide general guidelines for interpreting particular experiences, or performances which create as well as comment upon prior experiences’, but also function as ‘an aesthetic and moral form underlying clinical action. That is, therapists and patients not only tell stories, sometimes they create story-like structures through their interactions.’^43^ Pregnant woman are not sick, but their narratives can be seen within the wider context of illness narratives because they do share many of the same features, such as interactions with medical professionals and institutions, and sometimes unpleasant or traumatic interventions and practices. However, while some women saw maternity as a medical event, others did not, and this divergence formed an important feature of their stories and will be explored further below.

### 2.4 Narratives of war

Women’s wartime experiences also shaped their narratives of birth, however, and the war was an important trope. Examining how women (as opposed to men) told their war stories, Penny Summerfield found that ‘multiple discourses concerning women’s wartime lives were ‘taken up’ by women recounting their experiences, and were deployed by them in constituting themselves retrospectively as wartime women.’^44^ Certain types of narrative forms predominated, though, which often formed pairs of contrasting ways of telling the same story, such as the wartime discourse of the young woman as a ‘free agent at the disposal of the state’ in contrast to the pre-war discourse of the ‘dutiful daughter of dominant parent’, or the ‘idea of women’s heroic engagement in warfare’ as opposed to the image of women ‘stoically enduring the pressures and privations of a war waged by men’.^45^ In the remainder of the article I will examine how the war featured in the stories of pregnancy and birth of this generation of women. Through analysing the ways in which their tales of maternity were interwoven with wartime discourses I will explore how women incorporate such complex cultural tropes into their accounts.

## 3 Babies for the war effort

During the Second World War babies were, in the words of Virginia Nicholson, ‘very much wanted by the powers that be.’^46^ Interwar concerns about the declining birthrate became more pressing during the war. By 1939 the British birthrate had dropped to below replacement levels, with two million fewer under-fourteens than in 1914 with a worsening situation developing by 1941 when the figure of 579,091 was an all-time low.^47^ In response a pronatalist position was widely adopted by the government and others including labour organisations and members of the medical profession, labour organisations. Denise Riley defines this pronatalism as ‘that despondency and alarm over the low birth rate, both past and as anticipated by demographers, which took the solution to the problem to be encouraging women to have more children.’^48^ Riley notes that both the concern and the proposed remedy ‘had been building up throughout the 1930s and became more generally diffused towards the end of the war.’^49^

In this pronatalist context, having a baby was a way for women to contribute to the war effort. When the novelist Naomi Mitchinson’s daughter-in-law told her that she was expecting a baby at this time, Mitchinson’s reaction was: ‘It’s one in the eye for Hitler.’^50^ Helen Brook, born in 1907, was a London, housewife, mother and air-raid warden during the war. Interviewed by Mavis Nicholson about her life in the war she explained that after marrying her second husband in 1937 she found

I couldn’t become pregnant, which was very, very important to me, because I thought that any moment Robin was going to be called up. He hadn’t immediately become a soldier, because he was in a reserved occupation. I saw a specialist, Gladys Hill, from the Royal Free Hospital who was in Harley Street, just round the corner from us. And she said I had to have a little operation, like having my tubes blown, which in those days was considered fairly hazardous. So off I bravely went and had this job done, when she said to me, ‘Now go home and do your duty.’ So I had to rush off home and said to Robin, ‘Come on, it’s for England.’^51^

The belief that women should have babies for the national good endured in the years after the war. Women were expected to play an important role in post-war reconstruction through their roles as mothers. Julie Summers has noted how, ‘The government, women’s magazines and male commentators on child welfare put the onus for tackling the post-war years onto women. There was a sense that they held the key to making the future better for their men and children, that they had a duty of sacrifice in response to everything the men had fought for’.^52^ Pronatalist views that having children was a way for women to contribute to society remained influential. Rose was born and brought up in Yorkshire in the 1930s and had two children in 1959 and 1961. She recalled that, ‘after the war everybody was having children … I even had the thought, ‘Well I really should have children it would be selfish not to, you know you mustn’t leave it to everybody else, you must make some sort of contribution, you must all have some place in society’.

As well as being for the national good, maternity was a form of war service. Verily Anderson, born in 1915, was in the First Aid Nursing Yeomanry (FANY) between 1939 and 1941. In her autobiography of the war *Spam Tomorrow* she writes how she had volunteered for service abroad, hoping for a ‘dashing adventurous life’.^53^ However when her ambitions ‘came to nothing’ and she found herself stationed in London, she concluded that her future contribution to the war effort lay in marriage and motherhood. She reported that she ‘came home proud’ after her pregnancy was confirmed at the local antenatal clinic.^54^ Joy also exchanged the Services for motherhood. Born in Birmingham in 1923, she had joined the Women’s Royal Nursing Service after school. She married an airman and left the service after falling pregnant with her first child. Similarly to Verily Anderson, in her narrative she talked of her maternity as swapping one form of war service for another. She told me proudly that she only returned home four months before her son was born, adding, ‘I’m sure by today’s standards that was a bit less than desirable.’

While this pronatalism was the dominant trope for the wartime generation there were dissenters. Not all women agreed with the goal of increasing the population, although they also referred to pronatalist attitudes as the prevalent viewpoint, reinforcing its importance in popular discourse. Discussing the falling birthrate, Nora, a young midwife interviewed by Celia Fremlin, a Mass Observer in Kent in 1944, was critical of pronatalism: ‘It’s only because they want men to fight in the next war. The more babies people have, the more they’ll have to fight for them in the next war. I think it’s horrible. They don’t want the babies for their own sakes at all, just for wars.’^55^ Women could also be deterred from having children due to the war. Fremlin also spoke with Nora’s sister, Freda, a twenty-seven-year-old mother of two young children. Freda had been traumatised by her experience of an air raid and Fremlin reported that ‘The shock of the vent [bomb blast] colours most of Freda’s conversation. She says she wouldn’t dream of having any more children until the war is over.’^56^ Recalling the blast Freda said

The two of them were in their beds when it happened, and I rushed upstairs and found them all covered with glass—every inch of Billy’s cot, it was a mass of glass. It was a miracle he wasn’t killed, but he never had a scratch. He was right under the clothes you see. My, but it gave me a shock. I still keep trembling when I think of it, what might have happened. And then think, suppose it had been a tiny baby there, it would have been killed wouldn’t it, that’s certain. No, I’d never have another baby while this sort of thing’s going on.^57^

Mabel had both her children during the war, the first in Croydon in 1940 and her second in Oxford in 1945. When asked whether she worried about having her children during the war, she replied, ‘well you just had to get along with it.’ In fact she later said that when she had found out she was pregnant for a second time she had been unsure that she wanted another child, but was persuaded into having the baby by her husband. While she did not directly say she considered terminating her pregnancy (which was illegal at this time) her recollection of her ambivalence on discovering she was pregnant suggested that the uncertainties and privations of war had played more heavily upon her experience of maternity than she had initially recounted.

For other women who became pregnant during the war, though, the war featured as a backdrop to their stories, but was not a central theme. Having their children during the war was a personal choice not a public act. Maud was born in the village of Churchill Heath in 1921 and moved to nearby Milton-under-Wychwood when she got married to a farmer during the war. When asked whether having children in wartime was worrying, she replied: ‘No. No it was all part of life [laughing].’ Sarah, a teacher from Oxford, had her first child in 1940, at the beginning of the war. Like Maud, she said she was not worried about having a baby during the war, and said she enjoyed her pregnancy: ‘I was very well all during my pregnancy and very happy and the war didn’t really hit us until later on.’

During and after the war maternity was seen as a central part of being a woman. Women were expected to contribute to society through reproduction, both bearing and rearing children, in the role of housewife and mother. Concerns about the birthrate which had been growing during the 1930s peaked during the war and the years immediately after. The significance of these pronatalist discourses to women was seen in the way they used the idea of contributing to the national good through motherhood in their accounts. However their narratives indicate that not all women bought into the assertion that they should be having babies for the war effort, instead viewing pregnancy as a personal choice and a private act.

## 4 Lord Woolton’s ‘preggies’: the war and antenatal care

In order to encourage childbearing and support pregnant women the government promoted antenatal care. During the interwar years antenatal care had been developing along the models of infant welfare created before the First World War. Tania McIntosh reflects that motherhood became seen as ‘a matter of national significance, and regulation, whether through clinics or books of advice, helped to propel it from a private act to a public duty.’^58^ In July 1929 the Ministry of Health had issued a *Memorandum on Antenatal Clinics: Their Conduct and Scope* which specified the minimum scope and intervals for antenatal examination: a first visit at sixteen weeks followed by further visits at twenty-four and twenty-eight weeks, then fortnightly to thirty-six weeks and weekly visits thereafter. Visits would take place either at the clinic or in the patient’s home. During the visits the uterine height and girth was supposed to be taken, the fetal heart listened for, and the urine tested. It was anticipated that only the first examination and those at thirty-two and thirty-six weeks would be done by a medical officer, the rest being completed by midwives.^59^ Antenatal care continued to expand in the years leading up to the Second World War. The numbers of women attending antenatal clinics as a percentage of notified births grew from 38.9 per cent in 1932 to 48.4 per cent in 1936, and the number of local-authority clinics increased from 1060 to 1279.’^60^

However Oakley notes that renewed initiatives were taken during the war, ‘when central government undertook a quite unprecedented degree of responsibility for the national health. Two of its central strategies—evacuation and the rationalization of food supplies—concerned expectant mothers directly.’^61^ Norman Longmate argues that the Minister of Food, Lord Woolton, was particularly keen to promote the health of pregnant women due to his formative early years as a social worker in the Liverpool slums where he had seen for himself the effects of malnutrition in pregnancy: ‘His constant concern for his ‘preggies’ became something of a joke in the Ministry of Food, as did the photographs of bouncing infants regularly sent to him by proud and grateful parents with the ambiguous message, ‘Another of Lord Woolton’s babies’.^62^ Longmate has shown how the ‘tighter rationing became for the rest of the population, the larger the share that went to ‘the priority classes’,’ including pregnant women.^63^ The wartime experience therefore expanded the provision of antenatal care for women. While foodstuffs such as cod liver oil and cheap milk had been available in maternity clinics in more progressive towns before the war, the introduction of the welfare foods scheme in December 1941 extended these benefits to all pregnant women. A green ration book issued to pregnant women on the production of a medical certificate entitled them to receive orange juice, cod liver oil, vitamin A and D tablets, an extra pint of milk a day, an extra half ration of meat a week, and an extra egg per allocation.^64^ Women were urged by a Ministry of Food advertisement ‘Welcome Little Stranger’, that ‘The very best welcome you can give your baby is a beautiful body, a contented disposition—and a healthy, happy mother.’ Another advertisement warned them against giving these extra foods to other family members, telling them, ‘Don’t let Dad get all the meat.’^65^

The interviewees had differing stories to tell about their antenatal care. Recalling her experience with her first baby in 1945, Joy explained: ‘I went to antenatal once a month and everybody said that it was alright and then the happy time came, oh I couldn’t wait for it to happen’. She added, with satisfaction, that, ‘there wasn’t very much cosseting in childbirth then was there Celia.’ Joy had meant this as a rhetorical question, but Celia, who was being interviewed alongside Joy (they had been friends since childhood, both growing up, and still living, in Chipping Norton), did not want to share in Joy’s story and replied that, ‘I don’t know Joy, I wasn’t mixed up in it.’ Celia’s reluctance at having her experiences subsumed within Joy’s account signified that she had a very different story to tell. Celia was born in 1924 and had gone into nursing during the war. She had her first baby in 1952. The first difference between her story and Joy’s was that Celia’s baby was not born during the war, she was not ‘mixed up in it’. Hers was not a heroic story of battling war-time hardships. Furthermore, unlike Joy she said she did not enjoy being pregnant and her narrative centred on her ill-health. She recalled: ‘I had [my son] in the Radcliffe because unfortunately I was pre-eclamptic and I’d been in hospital here for two weeks I think, and I had been very, very sick, I was terribly sick all the time’.

As demonstrated by the case of Joy and Celia, there were differences between those women who saw pregnancy as a state or health or ill-health. For both groups their view was shaped by their attitudes towards the antenatal care they received. During the course of their interviews I encouraged the respondents to comment upon the differences between the experiences of their generation and those that went before and after. Discussing their antenatal care the interviewees reflected that they benefited from antenatal check-ups, which their mothers and grandmothers often had not received, but they also felt their care was minimal in comparison to that which their daughters or granddaughters enjoyed. They reported how they either made one or two visits to their doctor or the doctor or district nurse visited them. It is noteworthy that they did not recall receiving the number of visits that were prescribed in the 1929 memorandum. This divergence may be the result of a number of factors, such as misremembering, that they were ‘non-attenders’ and did not make use of the service on offer, or did not view their visits to antenatal clinics being a formal part of their care and only included appointments with their doctor or midwife. For example Enid, whose first baby was born in 1943, summed up the whole process: the district nurse would ‘come round, they’d come round and examine you, and the doctor would. That was about all that happened really. And then you’d be examined afterwards and that was it.’ Theresa, whose first baby was born in 1948 explained: ‘Well, you just informed the district nurse and she came once a month and took your blood pressure and found out if the baby was in the right position and that was it.’ Phyllis, who was living in Shipton-under-Wychwood and had her first baby in 1946 said: ‘Well in those days when you were pregnant the doctor only saw you at four months and eight months, that’s all the medical, you know, that’s the only time that he wanted to see you’. Madge, a resident of Milton-under-Wychwood, got married just before the outbreak of war in 1939 and had her first baby nine months later in 1940. Discussing her antenatal care she said: ‘Well yes the doctor that we had then was the father of the doctor that we’ve got now … and I went to him, but we didn’t have much care in those days … he only came in about twice during that time, and I wouldn’t know he was coming and he’d say, “Oh I’ve come to see how you’re getting on, go upstairs and lie on the bed”, you know sort of thing, and in about two minutes it was all over.’

The perception of the interviewees having their children in the 1940s and 1950s was that their antenatal care was limited in comparison to later generations. This belief indicates that the women felt pregnancy and childbirth were very different today and this difference was an important part of their stories. Implicit in their discussions of antenatal care, a further theme arose, namely whether pregnancy should be considered a medical matter or whether it was a natural part of a woman’s life. Some women contrasted their experiences negatively and indicated regret that they had not received more care. Tilly was born in 1924 in Lossiemouth in Scotland. A teacher, she had two children in the early 1950s. Comparing her antenatal care with that of women today she said, ‘What I think is marvellous now, is the scans. I do wish I could have had a scan.’ Other women were pleased they had not undergone the levels of monitoring and intervention that pregnant women experienced today. Cassie was born in 1913 and had worked in the War Office during the war. She had delayed having her first child, born in 1946, until the war was over, which meant she was already in her thirties when her daughter was born. She said, ‘in those days there weren’t all these worries about elderly pregnancies, and you know I didn’t have any tests, nobody told me about possibilities of Down’s Syndrome or what have you with older parents and thank goodness I didn’t [know]’. She added that she thought the attention women today received was ‘a mixed blessing.’ Discussing their antenatal care could also provide women with an opportunity to contrast the hardships of wartime and the immediate postwar period with today. Reflecting upon her experiences, Madge, whose five children were born between 1940 and 1948 recalled, ‘it was during the war and everybody was busy … well I just got on with it, I didn’t think too much about it, I think about it now because I think, compared with how we managed, they have so much help today.’

## 5 Childbirth as a heroic act

Significant changes took place in how women gave birth during the war. The growing use of institutions meant that many wartime women were the first among their kin to deliver their babies in hospital rather than at home, exposing them to a more medicalised model of care away from traditional support of family and friends.^66^ Although this trend towards hospital delivery had begun before the war and continued and strengthened in the years after, in wartime it took on a new context. Wartime pronatalism focused attention on maternal as well as infant mortality. An editorial in the *Times* in 1944 about a proposed national maternity service stated that in war ‘Every mother who dies in childbirth is a national loss.’^67^ Even before the outbreak of war, in its evacuation plans, the government prepared to open temporary maternity hospitals as soon as war broke out in requisitioned nursing homes and large houses, with expectant mothers being billeted in the district until they needed residential care.^68^ Pregnant women (along with children, the blind and crippled persons) were among those for whom evacuation from the vulnerable urban areas was considered a priority.^69^ While in actuality only about 13,000 expectant mothers were involved in the first evacuation, Longmate posits that the start of the war was probably the worst time to have a baby as maternity clinics and hospitals in the evacuated cities had been closed and the emergency hospitals in the country and on the coast were overcrowded.^70^ However bombing and rationing caused new difficulties in the years that followed. For example on 3rd May 1941 Mill Road Hospital in Liverpool was a victim of German bombing. The maternity ward was hit killing many mothers and their new babies.^71^

In the military-maternity analogy childbirth acts as a test of a woman’s character, a rite of passage, in the same way that going into battle is the test of a man. For women giving birth during the war this analogy became more was pertinent because, as Jennifer Purcell notes, during the ‘People’s War’ every act was potentially heroic.^72^ Throughout her account of the birth of her first baby Verily Anderson drew the link between maternity and military service. She directly compared her experience in the FANYs with her stay at a maternity hospital

We *mothers*, as we were in many cases prematurely called, slept in large dormitories and ate our meals at long trestle tables exactly as we did in the more fashionable F.A.N.Y.s. We were presided over by an attractive and extremely efficient young warden … I found The Barrens, on the whole, more interesting than the F.A.N.Y.s. The complex mechanism of our well-filled bellies was less dull to discuss than the engines of F.A.N.Y. ambulances. My companions were certainly more interesting, perhaps because all had, or had had, husbands or lovers.^73^

Anderson later described having to exchange her clothes for her hospital nightdress in the same way as men exchanged their civilian clothes for uniform: ‘I was welcomed by two midwives already wearing masks; and they handed me a clean calico nightgown. “Do the button up at the back until you’ve had your baby. Then you can do it up in front.”’^74^ Being able to reverse the nightgown after birth was like a badge of honour. She depicted the birth as one might describe a battle, writing: ‘I floated away from the disgusting scene, but strangely not beyond the sound of distant gunfire, followed by the more deliberate notes of a cuckoo in the garden. Then came a small, high, furious wail above it all; and I was suddenly fully conscious and able to feel something soft and wriggling against my knees. With absurd appropriateness a far-off air-raid siren sounded the all clear. “It’s a girl,” somebody said.’^75^ An air raid was also a pivotal moment in Helen Brook’s account of the birth of her third child in May 1943. She told Mavis Nicholson: ‘Those days in hospital were really awful. We had raids all the time. My baby was born in the basement and I was up in a ward far removed from her. It was really nerve-wracking.’^76^

The adversity of wartime featured prominently in the accounts of the Oxfordshire women who had given birth in the war. Sarah had her first baby in the Radcliffe Infirmary in 1940. The air raids at this time were a central theme, although in Sarah’s case it was the indirect rather than direct effects of the raids which were important. She explained

there were actual raids in 1940, yes there were … and the hospitals were evacuated from London so they were putting extra beds in the ward, and you never saw the same nurse twice, they were being whisked off to other places … it wasn’t terribly good … I was moved out of my bed down into the basement into a room that was a teaching room for the nurses, and they’d just pushed all the desks to one side and there were chalky blackboards with figures and things and statistics and whatever the nurses had last been using at their lecture still on the boards there, it was really filthy dirty, there was chalk and dust all over the floor and we had to go up and down, there was no lifts to that so we had to go up and down stairs so I was terribly glad to be out of it.

Olive, a teacher, was born in 1916 and had her first baby in 1945 in the Acland maternity home in Oxford. She also said the war had a detrimental effect upon her experience of giving birth. She felt she had a ‘mismanaged labour’ which she attributed to staff shortages. She said, ‘I suppose it was wartime and they didn’t have an awful number of nurses.’ In consequence she was in labour a ‘long, long time and the doctor was quite cross because they hadn’t called him in as they should have done.’ Olive thought her daughter’s difficult birth had then influenced her personality. ‘I mean she’d had a very difficult birth and … it seemed to me she was [affected by it].’

In contrast Mabel had a far more positive story to tell and felt she had benefitted from the developments in the wartime maternity service. She had moved to Oxford from Croydon in 1941 and had given birth to her second child in Ruskin College, Oxford, which had been requisitioned and used as a maternity hospital, in 1945. She said it was ‘very good.’ It was ‘all done out as a hospital up there. You wouldn’t have known it wasn’t.’ She added, ‘We had good food, we got looked after very well. It was excellent. You know I think I was lucky to have gone there.’ While Joy thought the war brought hardships—‘you didn’t get very much cosseting in 1945 I can assure you’—she told her story in an upbeat manner, indeed the backdrop of the war enabled her to present herself as someone strong and brave who could overcome any difficulties placed in her way. She said that after birth ‘you weren’t taken to a nice cosy bed.’ Instead she was told to ‘lie there’ and that

‘In the morning the doctor will come to stitch you.’ Well I did, I lay there until 11.30 the following morning [laughing] when Dr Steel came to put [in the] … necessary stitches. But I had some breakfast which I remember being some porridge minus any sugar and a piece of bread and margarine … [I remember] being in the ward which was full, there were babies being born all over the place … And it was all very jolly, apart from the fact that a great many of us were you know deprived of the fathers, there were a lot with the fathers away.

Enid was born in 1925 and her baby was born during the war in 1942 at home in Benson. It is interesting that the war did not feature in her birth story and this may have been because she had a home birth. Unlike the women giving birth in hospital, where they could see the effects of the war in terms of the buildings being used and the staff who were available, Enid did not witness any noticeable results of the war on her experience. While she said the birth of her child was ‘not very good, it was a couple of days and it was horrible’, this had nothing to do with the war. She added

My biggest worry was that it wouldn’t come, that it wouldn’t happen [laughing]. Yeah, no you didn’t know anything really, nothing at all, no. And course you had no gas and air, nothing to ease the pain, nothing at all. But the only thing was that when the midwife came she never left you. If you were all day and all night she was there with you all day and all night. So you weren’t left on your own like you hear they are, put in a room, can be a bit frightening I should think.

In a similar fashion to the way in which the women interviewed contrasted their antenatal care with that received by subsequent generations, Enid evaluated her experiences of birth with those of women today. While she felt there had been improvements in that pain relief was now available, she also thought aspects of the care provided had worsened with women no longer benefitting from the continuity of care that a personal relationship with their midwives had brought.

Women’s stories of birth also revealed their divergent attitudes towards birth as a normal part of the life course or a medical event. While, in the words of Ivy, who had her first baby in 1947, ‘you weren’t ill with a baby’, there were women whose childbirth narratives included medical emergencies. For these women childbirth, like war, shared the spectre of death. They were also stories of wartime medical advances. Discussing the decline in maternity mortality, and the role of the war within this, Loudon argues that in the late 1930s and during the first years of the war it is probable that sulphonamides were the most important factor. As the war progressed, however, the importance of sulphonamides diminished and other factors—improving maternity services, increased availability of blood transfusion, and better nutrition due to special supplements allowed under wartime regulations concerning food rationing—played an increasingly dominant role.^77^ These developments were reflected in the interviewees’ narratives.

While Joy had told a story of a problem free pregnancy and childbirth, her birth narrative reached a dramatic conclusion when it came to discussing the postnatal period. At three months old her son was having trouble feeding. She was advised ‘to put him on the bottle’ and was given something to dry up her milk, but

A few days after that I came to in the night with a pain in my left breast that exceeded anything in childbirth, it was absolutely ghastly. This was on a Sunday, so ultimately I pushed him around in the pram not knowing what to do, I finally went to the hospital and you couldn’t walk in on a Sunday, you had to pull the bell pole and the matron … she came to the door and said, ‘Yes?’ So I told her. She didn’t say come in or anything like that. She said, ‘Oh just go home and take some more Epsom salts and bind yourself up again in the towel, that will take care of it.’ So I did, and nothing happened, and this pain went on and on, and I had to fetch my grandmother. And I went to the doctor and he said, ‘Well I can either get your temperature down or deal with the pain, I can’t do both.’ … So I went off to the Radcliffe, you couldn’t take your baby with you, so I had to find somebody who would look after my three and a half month old baby, not my grandmother, not my mother, and off I went to the Radcliffe. And I was in the Radcliffe for seven weeks, and I had penicillin every three hours, eight times a day, day and night … I had been twenty-two for ten days when [my son] was born, so I wasn’t very old or experienced. Anyhow I had the penicillin and I was told by one of the sisters that I was very lucky to have it because I would have died of septicaemia if this had been a few years ago.

Olive also benefitted from the discovery of penicillin, however she told a less positive account of the new medical techniques that had been perfected during the war. She explained that during the birth she had

lost quite a lot of blood and they gave me a transfusion afterwards which was quite unusual in those days they didn’t have many of those it wasn’t so done, and it wasn’t properly matched, and they didn’t know it wasn’t properly matched … they thought I’d got an infection but it was really trying to cope with the wrong transfusion, so I kept on having, I was on M and B [sulphonamide antibiotics] and then on penicillin for quite a long [time], weeks, five weeks I was in hospital, and then when I got home I had a massive abscess … I wasn’t [better] until the January and she was born in the October, it was quite a do [laughing].

As with war stories, women’s narratives of birth could be stories of life and death. However while a woman like Joy constructed her account as a heroic story of survival against the odds, enabled by medical advancements, Olive’s narrative indicates other women were less certain about how to present experiences and did not feel they fitted into a simple narrative of progress.

The experience of war left an important medical legacy, and it has been argued that the wartime Emergency Medical Service set the pattern for the NHS introduced in 1948.^78^ Wartime trends in maternity care continued in the years that followed. For example the provision of orange juice and cod liver oil was maintained after the war, responsibility for their distribution being transferred from the Ministry of Food to the Local Health Authorities.^79^ The number of hospital births also continued to grow. The proportion of deliveries carried out in hospital was 63.7 per cent by 1954.^80^ However there were also new developments. For example, during the 1950s the role of local authority antenatal clinics declined and care given by GPs grew in popularity;^81^ pain relief became in labour became increasingly available at home as well as in hospital;^82^ and the number of GP attended deliveries fell.^83^ However, the women interviewed for this study did not present these changes in the health services as being pivotal in their narratives.^84^ The war’s legacy was felt in other ways, however, and the continued spectre of the war in British popular memory was seen in women’s post-war accounts of pregnancy and birth. Camilla, who was born in 1937 in Sheffield, had grown up during the war. Describing her birth experience she alluded to the war joking that the Churchill Hospital in Oxford where she had given birth had been a military hospital: ‘that’s what it was like [laughing], it hadn’t changed.’ Marjorie, who was born in 1931 in Southampton, had also grown up during the war. She had her first child in 1959. She said memories of the war, encouraged by pethidine, came back to her during the birth: ‘I can remember sort of talking nonsense half the night about all sorts of irrelevant things [laughing]. Her [the midwife’s] assistant was very, very good and stayed all night, and I must have driven her up the wall, ranting on, not ranting actually but meditating about, I can remember talking to her about clothes rationing. All the sort of things that came back from war years.’

The war featured in women’s narratives of birth in different ways. For some women, like Enid, who lived in a rural area and did not face its most immediate effects, such as being evacuated or air raids, it played a minimal role. Enid was delivered at home by a midwife in the same manner as her friends and family who had given birth before the war. Rather than being a war story, her narrative centred on the differences between what it was like to give birth in the 1940s and 2000s. However most of the women interviewed did present the war as playing a defining role in their experience of childbirth. Women talked of putting up with or overcoming the hardships the war brought in terms of unsuitable buildings or staff shortages. There were also accounts of benefitting from wartime advancements in medical knowledge such as the use of penicillin. Finally, telling their stories of childbirth also provided women with the opportunity to demonstrate their stoicism, bravery and wartime spirit, the qualities that had been required of them during the war years.

## 6 Conclusion

This analysis of British women’s accounts of maternity during the Second World War has revealed how war stories and birth stories share some similar features—such as the spectre of death or the encounter with the institution—which reflected the cultural association between maternity and military service at this time. Moreover, the wartime conditions women had experienced influenced how they articulated their sense of themselves. Informed by pronatalism and ideals of motherhood as being a woman’s contribution to the war effort or national good, women used these linguistic elements in their construction of their own narratives. However while the war was an important presence in wartime women’s stories of pregnancy and birth, it also featured in them in many different ways. For some women it was the defining characteristic of their narrative, for others it was very much in the background. Other types of narratives were also prominent in their testimonies. The theme of change over time was significant, with interviewees engaged in a process of comparing the different experiences of generations of women over the past seventy years. Linked to this was the question of pregnancy being a state of wellbeing or ill-health. In discussing the increased medical involvement in pregnancy and birth which had occurred during the postwar decades, the interviewees contemplated whether having a baby should be viewed as a medical event. Nonetheless, while this study has revealed the intricacy of wartime women’s stories, the military-maternity analogy does shed light on British women’s experiences of pregnancy and childbirth during the Second World War. Maternity was viewed by the government (and supported as such), and by many women themselves, as being part of their wider role as ‘domestic soldiers’. When constructing their narratives of maternity this image of the domestic soldier was as important to the wartime generation of women as the themes of medicalization or change over time. Indeed they did not necessarily recognise the introduction of the NHS as precipitating major change, indicating that ‘access’ to healthcare services was less important in their narratives than the wartime tropes. This analysis of their accounts therefore demonstrates how women draw on a range of complex cultural discourses, such as those of war, when articulating their stories of pregnancy and birth, which have often been overlooked in historical metanarratives of twentieth-century British maternity care.

## Footnotes

1 Smith (1996, p. 5).

2 Oakley (1984, p. 35).

3 Lewis (1980, p. 15).

4 Lewis (1980, p. 28).

5 Lewis (1980, p. 16).

6 Lewis (1980, p. 187).

7 Loudon (1991, p. 47).

8 Oakley (1984, p. 124).

9 Loudon (1992, p. 265).

10 Harris notes that many schools had to close for long periods, and there was widespread disruption of school health services, maternity clinics and all forms of non-acute medicine. (Harris, 1992, p. 26).

11 Longmate, 2002, Loudon, 1991.

12 Huston (1985, p. 153).

13 Morgenstern-Leissner (2006, p. 203).

14 Davis-Floyd, 1992, Morgenstern Leissner, 2006.

15 Grayzel (2002, p. 107).

16 Usborne (1988, p. 400).

17 Stokes (2000, p. 373).

18 Bock (1983, pp. 402–403).

19 Temkin (1999, p. 588).

20 Britain was the only country in the Second World War to conscript women into the war effort. (Sheridan, 2000, p. 2).

21 Purcell, 2010, Sheridan, 2000.

22 Lord Woolton, BBC broadcast, 8 April 1940 as cited in Purcell (2010, p. 99).

23 Purcell, 2010, Sheridan, 2000.

24 Malcolmson and Malcomson (2012, p. 5).

25 Purcell (2010, p. 87).

26 Purcell (2010, p. 99).

27 Interview recordings and transcripts are held by the author.

28 Agricultural Economics Research Institute Oxford (1944).

29 Field (2001, p. 103).

30 Interviewees were asked to give their class of origin and were roughly divided between the working and middle class. There are difficulties in defining women by class however the language of class was clearly influential upon their lives and is a useful analytical tool.

31 Summerfield (1998, pp. 1–42).

32 Abrams (2010, p. 106).

33 Abrams (2010, p. 106).

34 Davies (2001, p. 275).

35 Giles (1992, p. 242).

36 Portelli (1991, p. 50).

37 Bertaux-Wiame (1982).

38 Sangster (1998, p. 88).

39 Laura Tisdall has discussed the complexities of analyzing self-narratives and life-writing (her particular focus being on autobiographies), in Tisdall (2013, pp. 351–358).

40 Kleinman, 1988, Mishler, 1984, Frank, 1995.

41 Kleinman (1988, p. xiii).

42 Davies (2001, p. 268).

43 Mattingley (1998, p. 2).

44 Summerfield (1998, pp. 15–16).

45 Summerfield (1998, p. 16).

46 Nicholson (2011, p. 229).

47 Nicholson, 2011, Summers, 2013.

48 Riley (1983, p. 151).

49 Riley (1983, p. 151).

50 Naomi Mitchinson, as cited in Nicholson (2011, p. 230).

51 Helen Brook, as cited in Nicholson (1995, p. 93).

52 Summers (2013, p. 288).

53 Anderson (1956, p. 8).

54 Anderson (1956, p. 98).

55 Fremlin (2000, pp. 212–213).

56 Fremlin (2000, pp. 206–208).

57 Fremlin (2000, pp. 206–208).

58 McIntosh (2012, p. 54).

59 Oakley suggests that these guidelines were derived from information from hospitals and public health departments about the type of antenatal care currently provided rather than having any clinical basis. (Oakley, 1982, pp. 8–9).

60 Oakley (1982, p. 12).

61 Oakley (1982, pp. 11–12).

62 Longmate (2002, pp. 172–173).

63 Longmate (2002, pp. 172–173).

64 Longmate (2002, p. 173).

65 Longmate (2002, pp. 173–174).

66 However family and friends continued to play an important role. For discussion of role of kin see Davis (2011a).

67 “A Maternity Service.” *Times* [London, England] 13 June 1944: 5. *The Times Digital Archive*. Web. 7 Nov. 2013.

68 Longmate (2002, p. 57).

69 Oakley (1982, pp. 11–12).

70 Longmate (2002, pp. 57, 167–168).

71 http://www.bbc.co.uk/news/uk-england-merseyside-13210575. Accessed 7 Nov. 2013.

72 Purcell (2010, p. 3).

73 Anderson (1956, p. 101).

74 Anderson (1956, p. 113).

75 Anderson (1956, p. 115).

76 Helen Brook, as cited in Nicholson (1995, p. 95).

77 Loudon (1988, note 26, p. 203).

78 Webster (2002, pp. 6–12).

79 Oxfordshire MOH, 1954, p. 25.

80 Oakley (1984, p. 215).

81 Oakley (1982, p. 14).

82 McIntosh (2012, pp. 84–88).

83 McIntosh (2012, pp. 95–99). For a discussion in the changes in maternity care between the introduction of the NHS in 1948 the 1974 reorganisation of the health services and how these affected women’s experiences see Davis (2011b).

84 Davis (2011b, p. 401).
